# The Thickness of Surface Grafting Layer on Bio-materials directly Mediates the Immuno-reacitivity of Macrophages *in vitro*

**DOI:** 10.1515/biol-2020-0020

**Published:** 2020-05-03

**Authors:** JiangWei Xiao, Tao Huang, JiJie Hu, Fei Zan, ZhaoHong Liao, RuiCai Gu, Gang Wu, Hua Liao

**Affiliations:** 1 School of Materials Science and Engineering, South China University of Technology, Guangzhou,510641, China; 2 Department of Anatomy, Guangdong Provincial Key Laboratory of Medical Biomechanics, School of Basic Medical Sciences, Southern Medical University, Guangzhou, 510515 China; 3 Department of Orthopaedics and Traumatology, Nanfang Hospital, Southern Medical University; Guangzhou, 510515 China

**Keywords:** Immune response, Macrophage, Poly-phenoxyethyl methacrylate (PHEMA) brushes, Surface grafting

## Abstract

Introducing the surface grafting layers to regulate bio-compatibility and bio-function is an important step in the tissue engineering field. However, whether the thickness variation of the introduced biomimetic layer impacts the behavior of the adhered immune effector cells is yet to be dissected fully. In this study, we used a surface-induced atom transfer radical polymerization (SI-ATRP) method to synthetize and graft poly-phenoxyethyl methacrylate (PHEMA) brushes having different lengths on the glass substrates. Primary murine peritoneal macrophages were collected and cultured on the PHEMA brushes and we investigated the influence of polymer brushes having different lengths on macrophages phenotype and function. Our results demonstrated that the thicker brushes (200 nm and 450 nm) are superior to the thinner layers (50 nm) for macrophages survival, proliferation, cell elongation and migration. Moreover, the thicker brushes are more beneficial for macrophage’s activities and functions, presented by the increased production of M1-associated cytokines IL-6 and MCP-1, the elevated cell phagocytosis and the activation molecule F4/80 expression, and the reduced macrophages apoptosis in thicker brushes-sustained macrophages. Our data suggests that the thickness of the substrate grafting layer directly impacts macrophages recruitment and pro-inflammatory function, which is important in determining the intrinsic immuno-compatibilities of the surface modified-biomaterials and mediates material-host interactions *in vivo*.

## 1 Introduction

The immune response to the implanted materials is a major determinant of whether the scaffold is compatible and supports regeneration or undergoes a foreign-body reaction. Indeed, foreign materials unavoidably elicit a biological response when implanted *in vivo*. Therefore, the immune response must be carefully considered in the material design in order to avoid unwanted cell recruitment/attachment, heightened secretion of inflammatory cytokines, fibrous encapsulation, or chronic inflammation [1, 2].

The innate immune system plays a critical role in tissue development, homeostasis, and repair of injured tissues. Macrophages are among the first responders to tissue injury and are required for successful tissue regeneration [3, 4, 5, 6, 7]. Macrophages are also primary responders to foreign materials. When implanted *in vivo*, macrophages are able to detect physical properties of the implants. For example, material geometry can influence the ability of macrophages to adhere to a surface, phagocytose, or align with one another [8]. Sub-micron silicon particles induce a robust inflammatory response in naive bone marrow-derived macrophages by secretion of IL-1b and TNF-a due to disruption of the lysosomal machinery in the cells [9]. A ‘‘local shape’’ of implants at the initial point of contact determines whether macrophages will spread along or initiate phagocytosis [8, 10, 11, 12]. Furthermore, the surface properties of biomaterials directly impact the foreign body response [10, 13]. Therefore, it is important to understand the interaction between immune cells and the implanted biomaterials owning special surface physical properties.

Biomimetic molecules or structures conjugated onto a special substrate surface could possibly impact cell behavior in a very sensitive way. Furthermore, the signals from the substrate surfaces may be shielded by the grafting layer, and the thickness variation of the introduced biomimetic layer may impact the behavior of the adhered cells. Thus, it is possible that, the properties of the surface grafting layer of biomaterials may impact immune cell response. Cellular mechanics can be regulated both by substrate properties and through biochemical cues in immune cells. Like many other cell types, macrophages are able to detect mechanical properties of their substrate such as stiffness [14, 15, 16]. Soft PEG hydrogels prevent macrophage spreading and inflammatory gene expression *in vitro*. Stiffer hydrogels prime macrophages for a higher expression of IL-1b and IL-6 [15]. However, the interaction between macrophages with substrates modified by a biomimetic layer with various depths, is yet to be dissected fully.

The PHEMA brush was reported to be very stable, without decomposition or decrement in film thickness in phosphate buffered saline (PBS) for 20 days [17]. Excellent antifouling ability and high structural stability make PHEMA promising biomaterials for biomedical applications [18, 19]. Recently, we polymerized hydroxyethyl methacrylate (PHEMA) on a glass substrate via the surface-initiated atomic transfer radical polymerization (SI-ATRP) method, allowing us to control the thickness and composition of the polymer brush accurately [20]. To study whether the thickness variation of surface polymer layer on the substrate lead to behavior change of innate immune cells, the present study seeded the primary murine peritoneal macrophages on PHEMA brushes and explored the macrophages response to the variation of PHEMA thickness, which may provide valuable clues for the design and preparation of the optimal biomaterial surface modulation.

## 2 Material and methods

### 2.1 Preparation and characterization of the PHEMA brushes on glass surface

Before grafting PHEMA brushes, surface amination in an APTES aqueous solution (0.4%, v/v) for 20 min was immediately performed on a dry coverslip (10×10 mm) cleaned and hydroxylated by the piranha solution (98% H_2_SO_4_ and 30% H_2_O_2_, 7 : 3 v/v) at 90°C for half an hour. ATRP-initiator, BIBB, was first introduced on the coverslip by immersing it in an anhydrous DCM solution containing TEA (2%, v/v) and BIBB (0.04%, v/v) in a bottle. Then, 30 ml aqueous solution containing 1 ml HEMA (8.24 mmol), 22 mg CuCl (0.22 mmol), 200 μl PMDETA (0.96 mmol) and 45 mg sodium L-ascorbate (0.44 mmol) was charged into the bottle to initiate the polymerization of PHEMA at 50°C on the surface. The polymerization was terminated at 30 min, 150 min and 300 min getting various layers of PHEMA brush with different thickness on surface.

ATR-FTIR spectra were obtained on the dry coverslip with polymer brush by using a Bruker Vector 33 spectrometer in the scan range of 600–4000 cm^-1^. All spectra were averages of 32 scans measured at a resolution of 4 cm^-1^. The water contact angles were measured with a contact angle measuring device (OCA15, Data physics, Germany) by the sessile-drop method at 25°C on the dry coverslip with polymer brush. 2 μl deionized water was dropped on each sample, and the data were obtained by using the equipped camera. All angles were averaged from three independent measurements (a droplet on three positions respectively). The thickness of the PHEMA brush was measured by AFM (Asylum research, American) in a force mode according to the reference [1, 2]. A silicon AFM probe (Budget Sensors) with a spring constant of 0.3 N m^-1^ was used for AFM nanoindentation. The trigger deflection was set to 60 nm so that the probe could be completely pressed on the wet coverslip surface with the polymer brush. The brush thickness was calculated according to the equation Z= Z_1_-Z_2_, where Z is the brush thickness, Z_2_ is the position where the probe deflection reaches 30 nm, and Z_1_ is the position where the probe deflection increases.

### 2.2 Collection and culturing of primary murine peritoneal macrophages

Peritoneal macrophages were extracted by collecting ascites, then cells were cultured in tissue culture flasks with DMEM supplemented with 10% fetal bovine serum (FBS, Gibco), 100 units per ml penicillin, and 100 μg/ml streptomycin sulfate at 37°C and 5% CO_2_. The medium was changed after 2 h to isolate macrophages from the mixed population of cells in lavage fluid. To confirm that the acquired cells are macrophages, cells were identified by flow cytometry and immunofluorescence with representative F4/80 after 24 h of cultivation. Cells were detected with an Olympus BX51 fluorescence microscope (Olympus, Japan) or flow cytometry (BD, USA).

**Ethical approval**: The research related to animals use has been complied with all the relevant national regulations and institutional policies for the care and use of animals.

### 2.3 Cells proliferation and Live/Dead detection

Macrophage proliferation was quantified by the CCK-8 assay kit (Dojindo, Japan) following the manufacturer’s instructions at the predetermined time intervals of 2 h. For cell viability evaluation, macrophages were stained with a Live/Dead Viability/Cytotoxicity Kit (Invitrogen, USA) following the manufacturer’s instructions. The number of viable cells (green) on brushes was counted by the Image-Pro Plus 6.0 software (Media Cybernetic, Inc.MD, USA) and compared to cells on glass surface.

### 2.4 Microsphere phagocytosis and immunofluorescence detection

The macrophages culturing systems were treated with a 10 ul/ml Lumisphere (Lum, Beisite) for 3 h, washed with PBS twice and fixed with 4% paraformaldehyde, then permeabilization with 0.1% Trition-X 100. After being washed with PBS, cells were blocked and followed by staining with Anti-F4/80-PE (Abcam, USA). Cell nuclei were counterstained with 4’,6-diamidino-2-phenylindole (DAPI). Slides were viewed with an Olympus BX51 fluorescence microscope (Olympus, Japan).

For cytoskeleton protein immune staining, cells were fixed with cold acetone and incubated with rat monoclonal anti-alpha Tubulin (Abcam, USA), rabbit monoclonal anti-Vimentin antibody (Abcam, USA), PE-conjugated goat anti-rat IgG and FITC-conjugated goat anti-rabbit IgG (Santa Cruz) were used as secondary antibodies. Nuclei were counterstained with DAPI. Slides were viewed using an Olympus BX51 fluorescence microscope (Olympus, Japan). For results analysis, the ImageJ v1.42 software (National Institutes of Health, USA) was used to quantify the positive staining. 20 random sights of each group were selected to measure the positive area rate [(the total area of the positive staining/the total area of each sight) ×100%].

### 2.5 Cell migration test

Macrophages were cultured on the coverslip or PHEMA brush grafted coverslips. Linear scratches were made when cells were grown to approximately 70-80% confluence, and recorded the initial scratch distance as W1. After 24 h, the scratch distance was recorded as W2. The migration degree was evaluated by the following calculation: migration (%)=（W1-W2）/ W1 x100%.

### 2.6 qRT-PCR

Total RNA from macrophages was extracted using Trizol reagent (Invitrogen, USA). 1 μg of total RNA was used for reverse transcription (RT) with a commercially available kit (RevertAid First Strand cDNA Synthesis Kit, Fermentas，Ontario，Canada). Real-time polymerase chain reaction (qPCR) was performed in triplicate with an ABI StepOne Plus system (Applied Biosystems, Foster，USA) and a fluorescence-labeled SYBR Green/ ROX qPCR Master Mix kit (Fermentas，Ontario，Canada) for IL-6, TNF, MCP-1, MIP-1α, and with glyceraldehyde-3-phosphate dehydrogenase (GAPDH) taken as an endogenous control (primer sequences and sizes of amplicons are listed in Table 1). The results were analyzed with SOS2.1 software (Applied Biosystems，Foster，USA).

**Table 1:** Primer sequences for qRT-PCR

Genes	reference ID		Sequence	Amplicon size
IL-6	NM_031168.2	For	.. 5’ GGCAATTCTGATTGTATG 3’	208bp
		Rev	5’CTCTGGCTTTGTCTTTCT 3’	
TNF	NM_013693.3	For	.. 5’TTTCAAACAAAGGACCAG 3’	100bp
		Rev	5’GGATCATTTCCGATAAGG 3’	
MCP-1	NM_011333.3	For	. 5’GGGTCCAGACATACATTAA 3’	119bp
		Rev	5’ ACGGGTCAACTTCACATT 3’	
MIP-1α.	NM_011337.2	For	. 5’ CTGCCCTTGCTGTTCTTC 3’	154bp
		Rev	5’ CAAAGGCTGCTGGTTTCA 3’	
GAPDH	NM_008084.3	For	. 5’CAATGTGTCCGTCGTGGATCT3’	124bp
		Rev	5’GTCCTCAGTGTAGCCCAAGATG3’	

### 2.7 Western blot analysis

Protein extraction of cell lysate was performed according to the manufacturer’s protocol (KeyGEN，China). Protein concentrations were evaluated using a BCA assay kit (KeyGEN，China). Equal amounts of proteins were electrophoresed on 6-12% SDS-polyacrylamide gel and transferred to Immobilon P membrane (Millipore, USA). Membranes were blocked for 1.5 h at RT. The following antibodies were used for detection: rabbit anti-Caspase-3 (Bioss, Beijing, China); Mouse monoclonal anti-GAPDH (KANGCHEN, Shanghai, China). Primary antibodies were incubated overnight at 4°C. The membrane was then incubated for 1.5 h at RT with horseradish peroxidase conjugated goat anti-rabbit IgG (Fudebio, China) and goat anti-mouse IgG (Fudebio, China). After three washes in TBS-T, the protein bands were visualized by enhanced chemiluminescence (ECL) detection reagents (Applygen Technologic Inc., China). The immunoreactive band was detected by the ECL detection system (Protein Simple, USA), and densitometric values were analyzed with ImageJ v1.42 software (National Institutes of Health, USA). The relative expression of each immunoreactive band was calculated by comparison with GAPDH.

### 2.8 Statistical analysis

All data are expressed as mean ± standard deviation (SD). One-way ANOVA was used for multiple comparisons (Duncan’s multiple range test) using SPSS ver.13.0 software. *P* values<0.05 were considered as statistically significant.

## 3 Results

### 3.1 The thickness of PHEMA brushes on glass substrate impacts on morphology, proliferation and cell activity of macrophages

**Figure 1:**
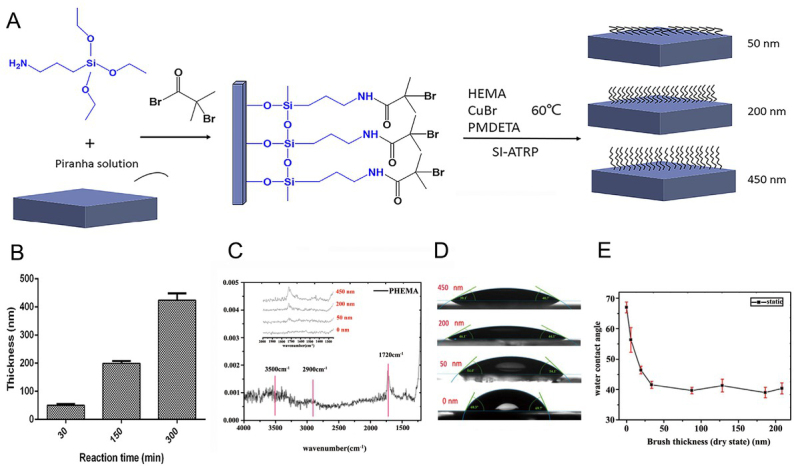
**Illustration of the measurement of PHEMA brush thickness on substrate. A** Experiment process of the PHEMA brush on a coverslip; **B** PHEMA brush thickness of different polymerization time by AFM; **C**. ATR-FTIR spectra of the PHEMA brush with different polymerization times; **D** Images of water contact angle on PHEMA with various thicknesses; **E** Static water contact angle on PHEMA brush with various thicknesses. Tests of c, d and e were conducted on dry sample coverslips

We prepared and grafted PHEMA brushes on coverslips by the SI-ATRP method. The thickness of the PHEMA brushes were about 50 nm, 200 nm and 450 nm, respectively (Figure 1).

**Figure 2:**
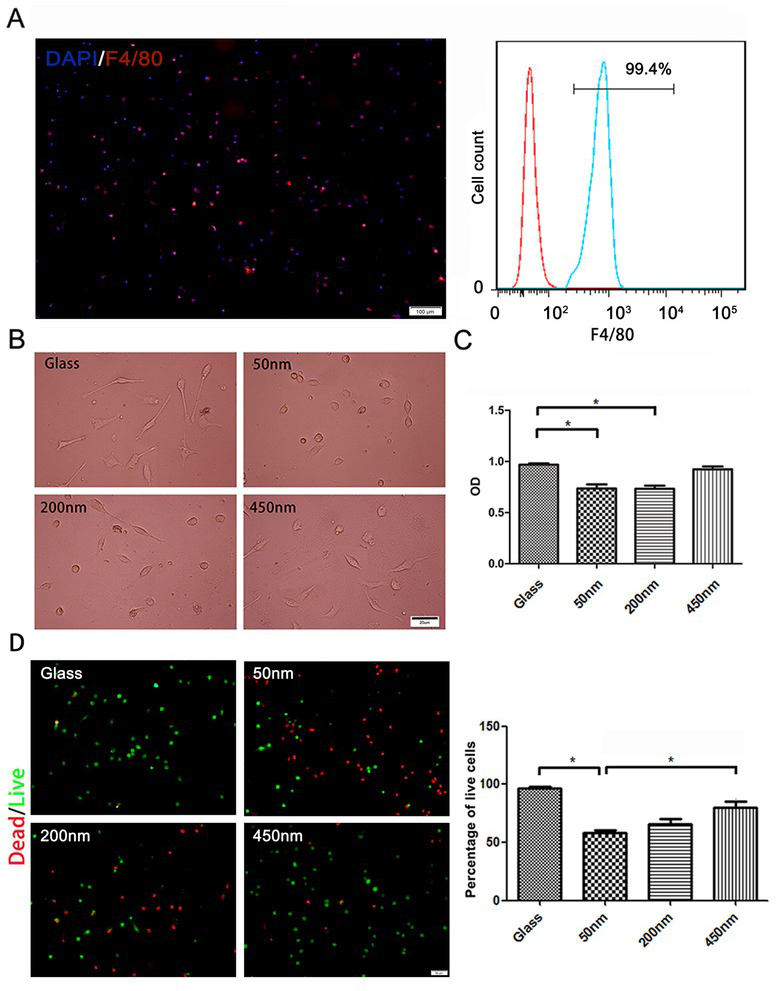
**Proliferation and cell activity of macrophages on the brushes with various depths A**. Macrophages were identified by flow cytometry and immunofluorescence with representative F4/80 after 24 h of cultivation. Bar = 100 μm; **B** Macrophages culture on the bare glass or on the brushes with various depths surface for 48 h. Bar = 20 μm; **C** Cell viability detected at 48 h by CCK-8 assay; **D** Live/dead staining of the macrophages after 48 h of cultivation. ANOVA was used for multiple comparisons. All data are presented as mean±SD (n≥3). (*p<0.05). Bar = 200 μm

There was an indication that the cells we received were characterized as macrophages (Figure 2A). As shown in Figure 2B, macrophages survived well on the bare glass surface, and cell spreading can be observed on glass surface after 2 days culturing. In contrast, the grafting of PHEMA brush onto the glass led to the alternations in macrophages shape and proliferation, presented by the lesser cell number and the shorter cell protuberances on the brush surface, comparing to that on glass surface. Interestingly, we found that the thicker brushes (200 nm and 450 nm) were better for the elongation of the unpolarized macrophages, than the thinner brush (50 nm). Using CCK8 test and cell live-dead staining, we further proved that, the thicker brushes promoted macrophages proliferation and increased cell activities, compared with the thinner brush, despite the highest cell proliferation appeared in cells survived on the bare glass surface (Figure 2 C, D). Our data thus suggested the direct interference of the brush depth for macrophages proliferation and activities.

### 3.2 The thickness of the grafting layer on glass substrate determines elongation and migration of macrophages

**Figure 3:**
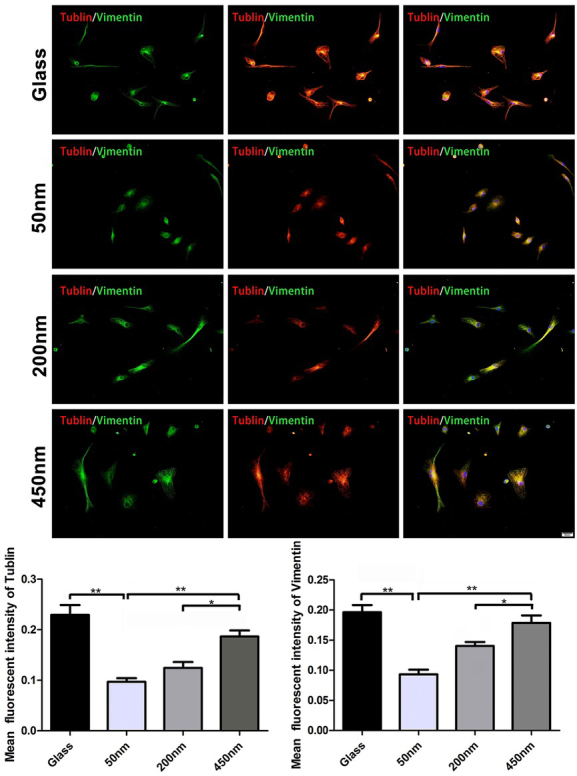
**The altered expression of cytoskeleton protein in the cultured macrophages on the brushes with various depths**. Representative Vimentin and Tublin immunofluorescence stained macrophages obtained after 48 h of cultivation. ANOVA was used for multiple comparisons. All data are presented as mean±SD (n≥3). (*p<0.05, **p < 0.01). Bar = 20 μm

**Figure 4:**
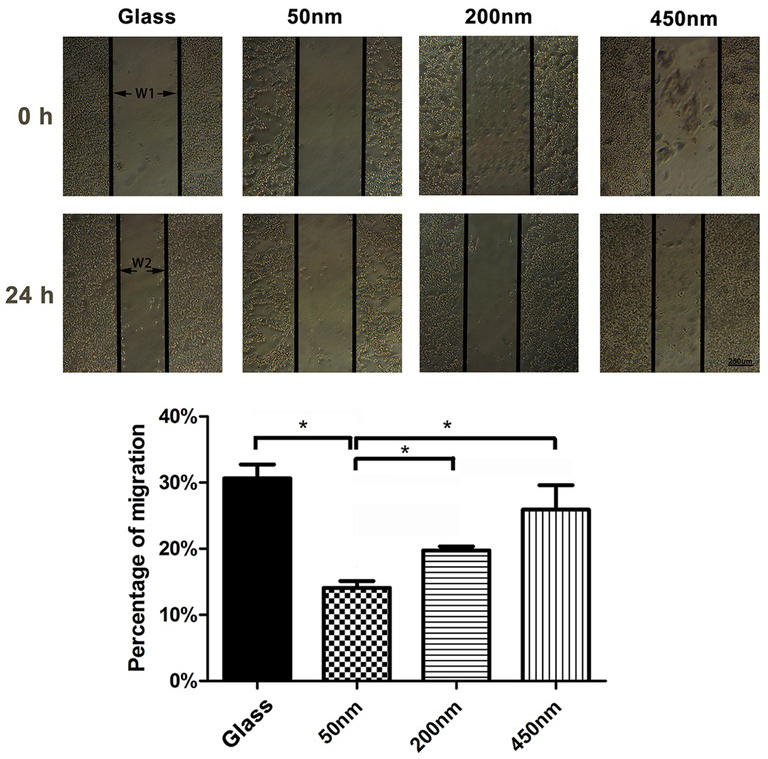
**The impact of brush depths on macrophages migration**. The migration of macrophages detected by scratch wound assay, migration (%)=（W1-W2）/ W1 x100%. ANOVA was used for multiple comparisons. All data are presented as mean±SD (n≥3). (*p<0.05). Bar = 250 μm

As shown in Figure 3, PHEMA brushes with different depths resulted in the altered expression of the cytoskeleton protein in the cultured macrophages. Through the cell scratch test, we further found that macrophages moved in a brush depth-dependent manner: cell spreading and migration on the thicker brushes (200 nm and 450 nm) were much more active than of that on the thinners (50 nm) (Figure 4). Giving that thicker brushes are favorable for macrophages migration, and the increased cytoskeleton proteins expression in cells along with the increase of brush thickness, which may account, at least in part, the depth of polymer brushes directly mediates macrophage attachment and motility.

### 3.3 The thicker brushes are more beneficial for macrophages recruitment and functions

**Figure 5:**
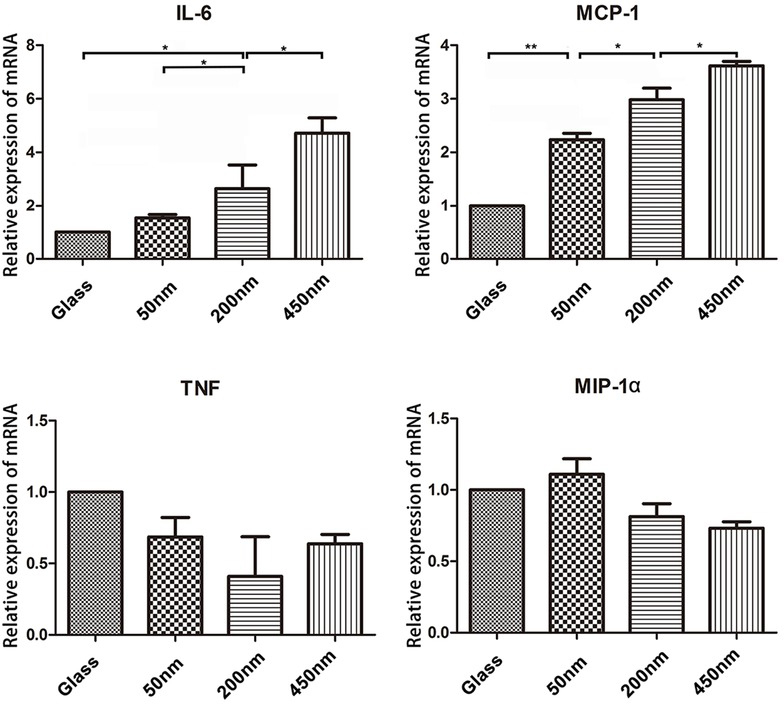
**Brushes with different depths selectively modulated the production of inflammatory mediators of macrophages**. mRNA levels of IL-6，MCP1，MIP-1α and TNF were quantified by qRT-PCR. One-way ANOVA was used for multiple comparisons. All data are presented as mean ± SD (n≥ 3). (*p < 0.05)

We found cells cultured on brushes with different depths selectively modulated the production of inflammatory mediators in cultured cells. As presented in Figure 5, in IFN-γ polarizing conditions, brush-sustained macrophages markedly up-regulated gene levels of M1-associated cytokines IL-6 and MCP-1. Indeed, 450 nm brush-sustained cells maintained a threefold increased gene levels for the two cytokines than glass-sustained cells. Instead, brush depths had no influence for other M1-cytokines production, involving TNF and MIP-1.

**Figure 6:**
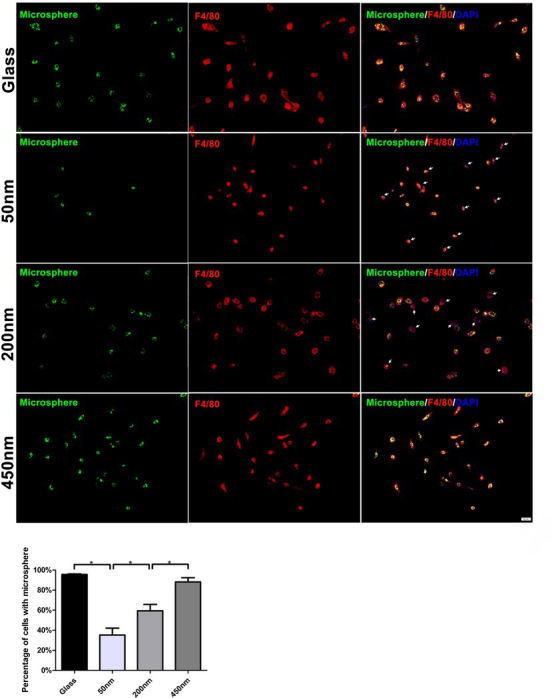
**The brush depths impact on macrophages phagocytosis ability**. Green microspheres were engulfed by F4/80^+^ cells. Cells without green microspheres were indicated by white arrows. One-way ANOVA was used for multiple comparisons. All data are presented as mean ± SD (n ≥ 3). (*p < 0.05). Bar = 20 μm

In Microsphere phagocytosis, we found cells that survived on thicker brushes (200 nm and 450 nm) captured more microspheres than cells on glass surface. In line with this, we observed the number of F4/80^+^ cell increased on thicker brushes, especially on the 450 nm brush, compared to that on glass surface. As expected, green microspheres were mainly engulfed by F4/80^+^ cell (Figure 6). Since F4/80 expression is a later marker for macrophage maturation and activation, these data indicate that the thicker brushes are superior to the thinner layers for promoting macrophages maturation and exerting immune function.

### 3.4 Macrophages cultured on thinner PHEMA brush are more readily suffered from apoptosis than on thicker ones

**Figure 7:**
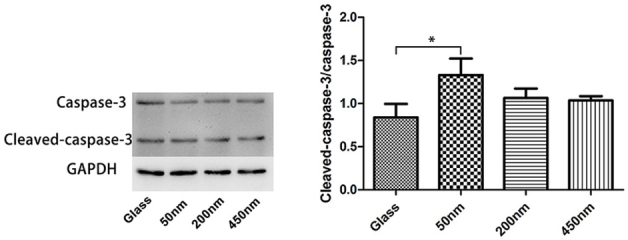
**Apoptosis of macrophages on the brushes with various depths**. Proteins expression of cleaved capase-3 and caspase-3 were showed in picture. One-way ANOVA was used for multiple comparisons. All data are presented as mean ± SD (n ≥ 3).

To assess whether the brush depths could directly lead to the programmedmacrophages death we examined protein levels of the cleaved or un-cleaved caspase-3, through western-blot analysis. Not surprisingly, we noticed that at the 48 h culturing point, the ratio of the cleaved capase-3 to caspase-3 was higher in macrophages on the thinner brushes (50 nm), than cells on the thicker ones. Instead, this ratio was similar in cells on thicker brushes (200 nm and 450 nm) compared to cells on glass surface (Figure 7). Combined, our result indicates that the depths of the grafting layer on the substrate are vital for the apoptosis initiation in polarized macrophages.

## 4 Discussion

Introducing or grafting molecules with biological activity onto biomaterial surfaces has been widely applied to regulate cell destination or increase material biocompatibility, because it is easy to be implemented on various types of biomaterials without distinct alteration of the bulk properties. These bioactive grafters and their interacting cellular receptors mediate the bidirectional reaction between the extracellular and intracellular compartments to influence cell growth, survival, differentiation, and morphogenesis [24, 25].

Monocytes and macrophages are primary responders to foreign materials, and the ability of these cells to interact with the material is greatly impacted by physical properties such as the shape and size, geometry, topography, and porosity and biochemical properties such as surface chemistry, ligand functionalization, and degradation mode [8, 9, 10, 11, 12]. Inflammatory cell interactions with surface modulation elements of biomaterials have become increasingly recognized in recent years. For example, grafting RGD reduces inflammatory gene expression by macrophages *in vitro*, and reduces the inflammatory response to non-degradable PEG hydrogels i*n vivo* [26, 27]. Coating polypropylene meshes with various types of extracellular matrices shifts the macrophage response from M1 to M2 and reduces the foreign body response [28]. Formulations with surface-assembled poly(I:C) on PLGA(tt) microspheres readily activated immune cells with respect to the expression of maturation-related surface markers, pro-inflammatory cytokine secretion and directed migration [29]. However, few previously reported studies reveal whether the signals from the substrate surfaces can be shielded by the grafting layer, and whether the thickness variation of the introduced biomimetic layer impacts the behavior and functions of immune cells.

In this study, we polymerized PHEMA on a glass substrate via the SI-ATRP method, allowing us to control the thickness and stiffness of the polymer brush accurately. Murine macrophages seeded on the PHEMA brushes were used to address the sensibility of immune cells to feel environment changing, and to further investigate whether thickness variation of the surface grafting layer of bio-materials is a key factor in regulating immune cells behavior and function. Our previous reports suggested that precursor chondrocyte cultured on the substrates with short PHEMA brushes developed the spread morphology, but cells on long PHEMA brushes displayed a more rounded shape [20]. However, in contrast to the results of chondrocyte line, here, our data showed that the thicker brushes (200 nm and 450 nm) were better than the thinner ones (50 nm), for macrophages adhesion and survival, proliferation and cell activities. Since PHEMA is bio-compatible, and it is impossible to be degraded during a 48 h culturing period, the higher activities of macrophages on the thicker brushes can’t be explained by PHEMA induced immune toxicity. According to the tensional integrity theory, cells adjust their own tension to match the elastic modulus of ECM to reach equilibrium [30]. These physical signals are transduced into the cells through the ECM–cytoskeleton axis system to induce cell proliferation, adhesion, differentiation and exert biological functions. It is apparent that macrophages are prone to maintaining their phenotype on the surfaces having PHEMA brushes with long length. Despite the mechanism of the impact of brush length on macrophages response not being clear, this data indicates the direct interference of the depth of material-surface grafting layer on immune cells activities. The motility of immune cells surrounding implants can be greatly affected by material geometry and topography properties, and macrophages can detect the ‘‘local shape’’ at the initial point of contact that determines whether they will spread along the material. It was reported that, parallel gratings made of different polymers ranging from 250 to 2000 nm promote spreading and elongation of unpolarized macrophages [31]. Electrospun fibers, which can create scaffolds mimicking natural extracellular matrix, support differing amounts of macrophage response based on diameter, packing, and alignment features [32, 33, 34]. Through analyzing cytoskeleton molecules in macrophages cultured on PHEMA brushes with different depths, and by observing the migration state of these cells, we outlined that the depth of polymer brushes directly mediates macrophage attachment and motility.

The physical properties of bio-materials can also greatly affect macrophages function. Pathophysiologically, macrophages use the size of microbes they encounter to dictate the immune response they signal. A spherical diameter around 1.5 mm is superior to smaller spheres across a wide range of materials (including alginate hydrogels, stainless steel, and glass) for reducing macrophages response, perhaps due to reduced cell attachment to the particle surface [8]. Sub-micron silicon particles induce a robust inflammatory response in macrophages by the secretion of IL-1b and TNF-a [9]. Macrophages are most able to phagocytose microspheres around 2mm in diameter, both by the number of microspheres internalized, and their total volume [10]. A wide range of shapes can be engulfed by macrophages if the initial orientation is correct and total size primarily affects completion of phagocytosis. Further, the sub-micron size of particles can determine whether a particle is engulfed by endocytosis, a distinction that could impact the extent of macrophage activation [12]. Of note, we observed gene levels of M1-associated cytokines IL-6 and MCP-1 markedly up-regulated in the thicker brushes (200 nm and 450 nm)-sustained macrophages, yet have no impacts on other M1-cytokines production, involving TNF and MIP-1α suggesting the diverse sensibility of immune cells in the different cytokine gene initiation cascade in the feeling substrate grafting layer. When we turned to probe macrophages phenotype and phagocytosis ability, just as expected, we further demonstrated that the thicker brushes are superior to the thinner layers for promoting macrophages maturation (F4/80 expression up-regulation) and activation (the increase of fluorescence micro-particles engulfment). Macrophages are reported to be able to detect the mechanical properties of the substrate such as stiffness [14, 15, 16]. Soft PEG hydrogels (modulus of 130 kPa) prevent macrophage expression of inflammatory genes *in vitro*. Stiffer hydrogels (up to 840 kPa) prime macrophages for a higher secretion of IL-1b and IL-6 [15]. It has been speculated that brushes with a shorter chain length (low-depth brush) possess fewer conformations via chain rotation, and exists rigid polymer properties, otherwise, brushes with a longer chain length (high-depth brush) possess soft properties [20]. A decrease in the inflammatory response with decreasing material stiffness applies to even softer substrates such as polyacrylamide hydrogels below 150 kPa [14]. However, our data highlighted that macrophages are more adaptable to the softer thick brush surface, and thus elevates their activities and function, which implies that the bio-materials covered by the thicker surface grafting layer may induce the more severe inflammatory response *in vivo*. The surface properties of bio-materials also impact the macrophages fate, for example, hydrophilic or anionic surfaces causing macrophage apoptosis and inhibiting macrophage fusion *in vivo* compared with unmodified, cationic, or hydrophobic surfaces [13]. By exploring the cleaving degree of capase-3 in macrophages, we confirmed that the interactions with thicker brushes (200 nm and 450 nm) were in favor for macrophages survival and inhibiting cell apoptosis. This result further supports that, the thicker and softer grafting layers promote macrophages to exerting immune functions.

## 5 Conclusion

We show herein that macrophages can feel thickness changes of material grafting layers, and thus alter cell biological response to bio-materials. The thickness of grafting layers may directly impact on immune cell recruitment and function, mediate material-host interactions, and finally determine the intrinsic immuno-compatibilities of the modified bio-materials. Given that the thickness of the grafting layer impact phenotype and function of innate immune cells, it is likely to improve biomaterial-mediated tissue repair efficiency, by surface modulating to avoid unwanted immune cell recruitment, attachment and pro-inflammatory functions.
